# Emergency Physicians’ and Nurses’ Perspectives on Transgender, Intersexual, and Non-Binary Patients in Germany

**DOI:** 10.5811/westjem.20919

**Published:** 2024-10-29

**Authors:** Torben Brod, Carsten Stoetzer, Christoph Schroeder, Stephanie Stiel, Kambiz Afshar

**Affiliations:** * Hannover Medical School, Department of Emergency Medicine, Hannover, Germany; † Hannover Medical School, Institute for General Practice and Palliative Care, Hannover, Germany; ° These authors contributed equally.

## Abstract

**Introduction:**

Providing appropriate healthcare for transgender, intersexual and non-binary (TIN) individuals remains a significant challenge, as this group experiences higher rates of health inequalities, discrimination, and barriers to accessing care. Emergency physicians (EP) often lack formal training and knowledge about caring for TIN patients, while comparatively less evidence is available for other healthcare professionals, including emergency nurses (EN). Therefore, our goal in this study was to explore the experiences, knowledge, and attitudes as well as education/training needs of both ENs and EPs in Germany regarding the care of TIN patients.

**Methods:**

In February 2023, we electronically surveyed EPs and ENs from emergency departments (ED) across Germany. The survey, developed through literature review and collaboration with experts and members of the TIN community, consisted of 15 closed-ended items divided into three sections: experiences and knowledge; attitudes; and education/training needs. We used standard descriptive statistics and tested for group differences using the chi-square test.

**Results:**

Of the approximately 1,665 EPs and ENs contacted, 502 completed the survey and were eligible for further analysis (30% response rate). Of the respondents, 233 (46%) were EPs and 269 (54%) were ENs, with ENs being significantly younger and with fewer years in practice. More than half reported experience caring for TIN patients (71% of ENs vs 61% of EPs; *P* = 0.002), but there were significant gaps in medical and non-medical knowledge. Attitudes toward TIN patients were generally positive, but differences in communication approaches were noted, with ENs significantly more likely than EPs to limit their communication with TIN patients to what was necessary (25% of ENs vs 17% of EPs; *P* = 0.006). Most respondents (55% of ENs and 58% of EPs) had no training in the management of TIN patients, with only 8% of EPs and 17% of ENs having received such training during their medical/nursing school education (*P* = 0.01). Both groups agreed that there is an urgent need to increase awareness of emergency medical care for TIN patients among ED staff.

**Conclusion:**

Both emergency physicians and nurses in Germany demonstrated deficits in knowledge of and clinical preparedness to care for patients in the ED who identify as transgender, intersexual and non-binary, indicating a clear need for enhanced education, training, and institutional support to improve emergency care for this vulnerable patient population.

Population Health Research Capsule What do we already know about this issue?

*Transgender, intersexual and non-binary (TIN) individuals face significant health disparities and barriers to care in multiple settings, including EDs.*

What was the research question?

*To explore the knowledge and attitudes of emergency nurses (EN) and physicians (EP) in Germany regarding the care of TIN patients.*

What was the major finding of the study?

*ENs and EPs in Germany lacked key knowledge about TIN emergency care and had different approaches to communication with these patients*.

How does this improve population health?

*Recognizing the need to improve education and training for EPs and ENs regarding the care of TIN patients could lead to more equitable and respectful emergency care.*

## INTRODUCTION

Providing appropriate healthcare to people with transgender, intersexual, and non-binary (TIN) identities remains a significant challenge in many healthcare systems, including in Germany.^1^ In the United States and Europe, approximately 0.6% of the adult population identifies as TIN, whereas the prevalence among adolescents is estimated to be approximately 1.4–4.1%.^2^ ^,^ ^3^ ^,^ ^4^ While much of the relevant literature focuses on LGBTQ+ (lesbian, gay, bisexual, transgender, questioning/queer) patients in general and does not distinguish between sexual minorities (eg, LGB) and gender minorities (eg, trans), studies have consistently shown that TIN individuals in particular experience health inequalities, including higher rates of substance use disorder, disability, and mental illness.^5^ ^,^ ^6^

Their lifetime risk of attempting suicide is more than 10 times higher than that of the general population (54% vs 5%).^7^ Individuals identifying as TIN are also disproportionately affected by homelessness, underemployment, and extreme poverty.^8^ ^,^ ^9^ These challenges are compounded by barriers to accessing healthcare in both the US and Europe, as demonstrated for Germany in the InTraHealth study. Due to fear of discrimination or past negative experiences, many TIN individuals avoid interactions with healthcare professionals, which can lead to delayed presentation, diagnosis, and therapy.^6^ ^,^ ^10^

A recent study by Samuels et al found that nearly half of trans and non-binary individuals surveyed avoided care in the emergency department (ED) due to anticipated discrimination, fear of mistreatment, and long wait times.^11^ Deficits in knowledge and clinical preparedness have been demonstrated for emergency physicians (EP) and residents in the US and Canada. While most EPs (88%) provide care to members of this population, few (27.5%) have had formal training and, therefore, lack clinical knowledge about important aspects of caring for TIN patients.^12^ Similar deficiencies have been demonstrated among emergency medicine (EM) and pediatric residents.^13^ ^,^ ^14^ The specific situation in Europe and especially in Germany remains largely unexamined.

Little evidence is available for other healthcare professionals, including registered nurses.^15^ Emergency nurses (EN) are often the first to encounter these patients and to obtain relevant data, including gender. Receiving reassuring care at the beginning of emergency care is crucial to the patient’s sense of trust and safety and may mitigate negative feelings due to possible past mistreatment. While nurses appear to have a wide range of attitudes, knowledge, and beliefs that affect the care they provide to sexual- and gender-minority patients in general, they often lack LGBTQ+ education specific to the needs of this population.^16^ As of March 2024, to the best of our knowledge, there has been no published data on ENs and their attitudes toward TIN patients specifically. Despite increasing awareness and advocacy for sexual and gender minorities, until recently many health professional curricula (eg, medical school, nursing school) provided little formal instruction, presumably leading to the observed gaps in clinical and cultural competency.^17^ ^,^ ^18^

Most of the current literature on TIN emergency care comes from the US and Canada, with comparatively less research in Europe and no data available from Germany. Nevertheless, the perception that emergency care professionals know little about TIN individuals and their specific healthcare needs is also widespread among the TIN community in Europe; this is a concern for both healthcare professionals and patients.^4^ ^,^ ^19^ Therefore, our aim in this study was to assess the knowledge and attitudes as well as education and training needs of both EPs and ENs in Germany regarding the care of TIN patients.

## METHODS

### Study Design

We conducted a cross-sectional study of staff members of EDs in Germany using a web-based survey. The survey was distributed to 70 randomly selected EDs of all sizes across Germany. Both EPs and ENs were considered eligible to participate. The Ethics Committee of the Hannover Medical School approved the study (01/17/2023; No. 10706_B0_K_2023).

### Survey Development and Administration

We conducted a literature review of healthcare professionals’ experiences with TIN patients and the healthcare needs of this population. Based on existing studies and applied questionnaires, we developed a survey consisting of both new and previously used items to assess EPs’ and ENs’ experiences, attitudes, education and training needs related to caring for TIN patients.^12^ ^,^ ^20^ ^,^ ^21^ For this purpose, previously used items from the literature were prioritized and adapted for content, structure, and language in collaboration with an interdisciplinary group of experts in questionnaire development. In addition, we worked with members of the TIN community to develop new items to better address the specific needs and concerns of this population with regard to emergency care.

We pre-tested all selected items in our survey for clarity with six EPs and five ENs, similar to the intended study population and piloted the same electronic delivery method as planned for wider survey distribution using an anonymous online survey (SoSci Survey GmbH, Munich, Germany).^22^ The final survey consisted of 15 closed-ended items equally divided into three thematic categories: 1) five questions about the healthcare professionals’ experiences and knowledge; 2) five regarding their attitudes; and 3) five questions related to education/training needs, with response options presented as either single choice or 4-point Likert scales (strongly agree – somewhat agree – somewhat disagree – strongly disagree). Study participants’ demographic parameters were collected before the survey began, following consent for data use.

The anonymous, self-administered online survey was available for completion during a four-week period in February 2023. Invitations were sent via e-mail to ED medical directors with the request to distribute the link to the survey to their staff members, as no publicly available mailing list existed that allowed a direct invitation of EPs and ENs in these EDs. We estimated the number of EPs working in the 70 EDs to be approximately 742 and the number of ENs to be ≈923, according to public health data for EDs in Germany and recommendations of German EM societies.^23^ ^,^ ^24^ No reminders were sent. All participants who met the inclusion criteria and completed at least the demographic parameters and the content category on experience were included in the final analysis.

### Primary Data Analysis

We extracted raw data from SoSci Survey into IBM SPSS Statistics version 27 (IBM Corp, Armonk, NY) and calculated standard descriptive statistics, including frequencies. The chi-square test was applied for group comparison. We considered *P* < 0.05 to be significant. The effect size of the Cramer V is interpreted as high (V = 0.5), moderate (V = 0.3), and low (V = 0.1).

## RESULTS

During the study period, 536 surveys were returned, of which 502 were eligible for further analysis. Of those, 34 had to be excluded because the respondents did not complete at least the demographic parameters and the content category on experience. Ninety-five percent of respondents completed all 15 items. The overall response rate was 30%, with approximately 923 ENs (response rate 29%) and 742 EPs (response rate 31%) contacted by their respective ED medical directors to participate in the study. Of the respondents, 233 (46%) were EPs and 269 (54%) were ENs. There were significant intergroup differences in all demographic variables analyzed (sex, age, years of work experience), except for the size of the city where their ED was located. In particular, ENs were significantly younger (39% of ENs ≤ 30 years vs 23% of EPs ≤ 30 years, *P* = 0.001; V = 0.196) and had fewer years of work experience than EPs. Table 1 summarizes the characteristics of the respondents.

**Table 1.** Demographics and descriptive characteristics of emergency department staff who participated in the survey.

Demographics	Physician		Nurse		*P* < 0.05
	n	%	n	%	*P*
Total	233	46.4	269	53.6	
Sex					
Male	128	54.9	83	30.9	
Female	103	44.2	186	69.1	
Diverse	2	0.9	0	0	<0.001
Age group (years)					
≤20	0	0	6	2.2	
21–30	54	23.2	99	36.8	
31–40	81	34.8	80	29.7	
41–50	59	25.3	43	16.0	
>50	39	16.7	41	15.2	0.001
Size of city of workplace					
<20,000	15	6.4	25	9.3	
20,000–100,000	59	25.3	73	27.1	
>100,000–< 1,000,000	125	53.6	144	53.5	
≥1,000,000	34	14.6	27	10.0	0.31
Working experience (years)					
In training	0	0	16	5.9	
<5	78	33.5	51	19.0	
5–10	38	16.3	60	22.3	
>10	117	50.2	142	52.8	0.001

### Experience and Knowledge

**Figure 1.**
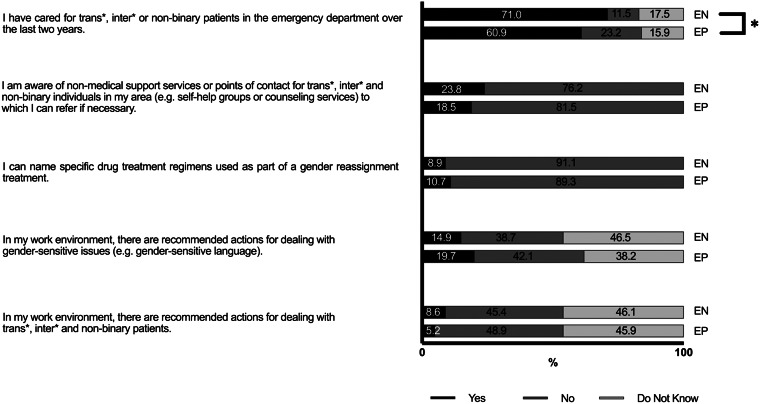
Experience and knowledge of emergency physicians and nurses in Germany in caring for transgender, intersexual and non-binary patients. * = *P* < 0.05. *EP*, emergency physician; *EN*, emergency nurse.

More than half of the respondents reported experience in caring for TIN patients in the ED in the prior two years. Compared with EPs, a higher proportion of ENs reported this experience (71% vs 61%; *P* = 0.002; V = 0.155) (Figure 1).

Both EPs and ENs lacked medical knowledge and knowledge of non-medical support services for transgender, intersexual and non-binary (TIN) individuals. While 24% of ENs and 19% of EPs were aware of non-medical support services or contact points for TIN individuals to which they could refer these patients, only 9% of ENs and 11% of EPs could name specific medication regimens used as part of gender reassignment treatment. In their work environment, almost all ENs (92%) and EPs (95%) reported a lack of official recommendations or their own limited awareness, of such recommendations for dealing with TIN patients in the emergency department.

### Attitudes

**Figure 2.**
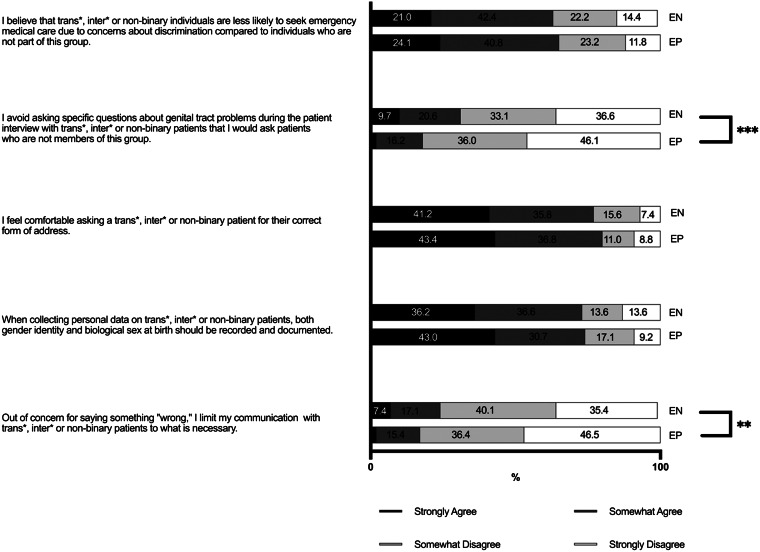
Attitudes of emergency physicians and nurses in Germany regarding transgender, intersexual and non-binary patients. ** = *P* < 0.01, *** = *P* < 0.001. *EP*, emergency physician; *EN*, emergency nurse.

Most respondents in both groups agreed that both gender identity and biological sex at birth should be documented when collecting personal information of patients presenting to the ED (73% of ENs and 74% of EPs) (Figure 2). Correspondingly, 77% of ENs and 80% of EPs felt comfortable asking TIN patients for their correct form of address. There were significant differences between ENs and EPs as to whether they limited their communication with TIN patients to what was necessary out of concern for saying something wrong (25% of ENs vs 17% of EPs; *P* = 0.006; V = 0.160). The EPs were also less likely to agree that, when interviewing TIN patients, they avoided asking questions about genital tract problems that they would ask non-TIN patients (30% of ENs vs. 18% of EPs; *P* = 0.001 V = 0.187). In terms of oppression awareness, the majority of both groups agreed that they believed TIN individuals were less likely to seek emergency medical care than non-TIN individuals because of concerns about discrimination (62% of ENs and 65% of EPs).

### Education and Training Needs

**Figure 3.**
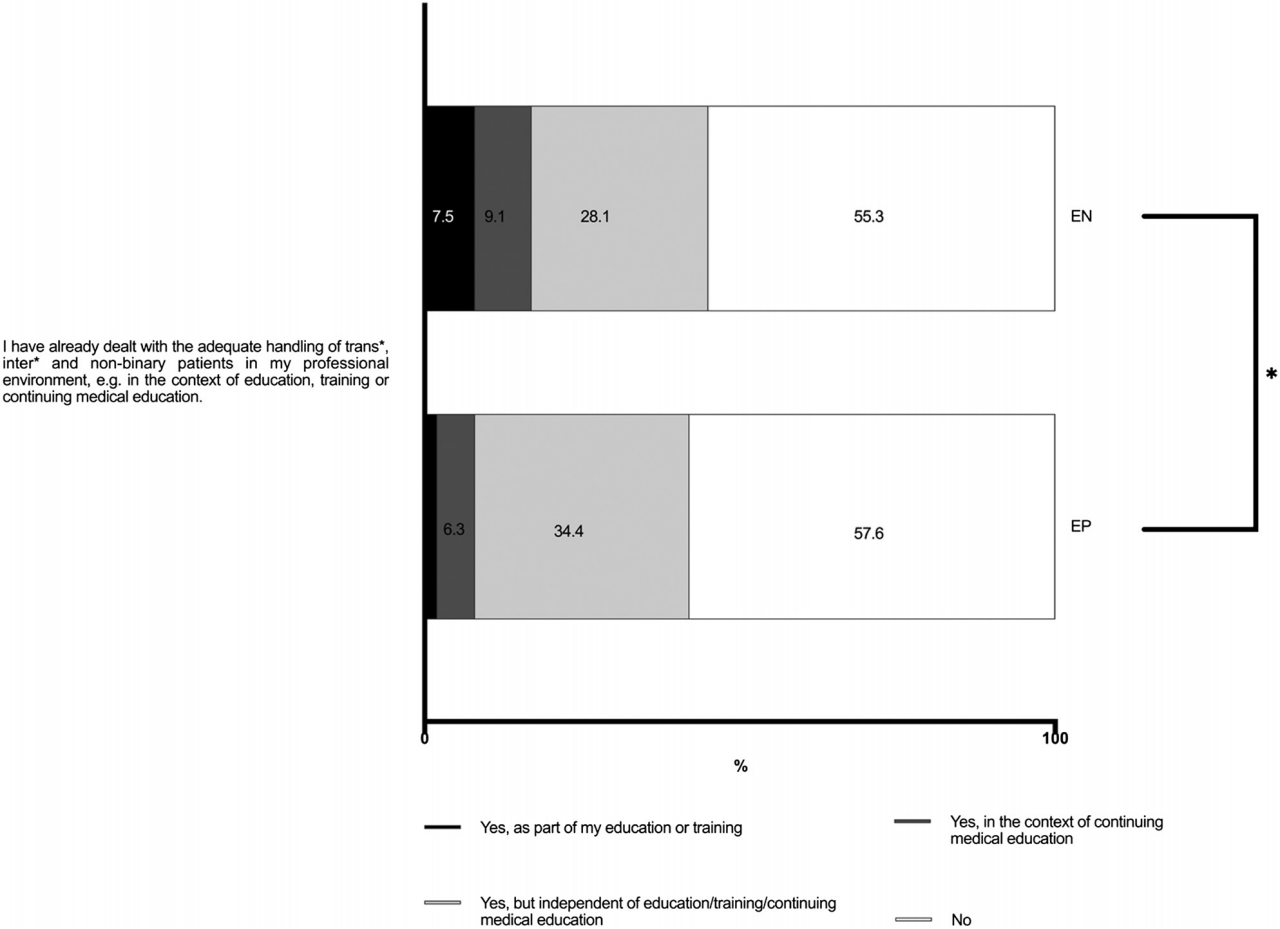
Education and training of emergency physicians and nurses in Germany regarding the care of transgender, intersexual and non-binary patients. * = *P* < 0.05. *EP*, emergency physician; *EN*, emergency nurse.

The majority of respondents reported that they had not received any training in the appropriate management of TIN patients (55% of ENs and 58% of EPs). Specifically, only 8% of EPs received such training during their undergraduate education (medical school) or postgraduate (residency/fellowship) training, while 17% of ENs received formal training (*P* = 0.01; V = 0.151). We found that 28% of ENs and 34% of EPs reported learning about this topic on their own (Figure 3).

**Figure 4.**
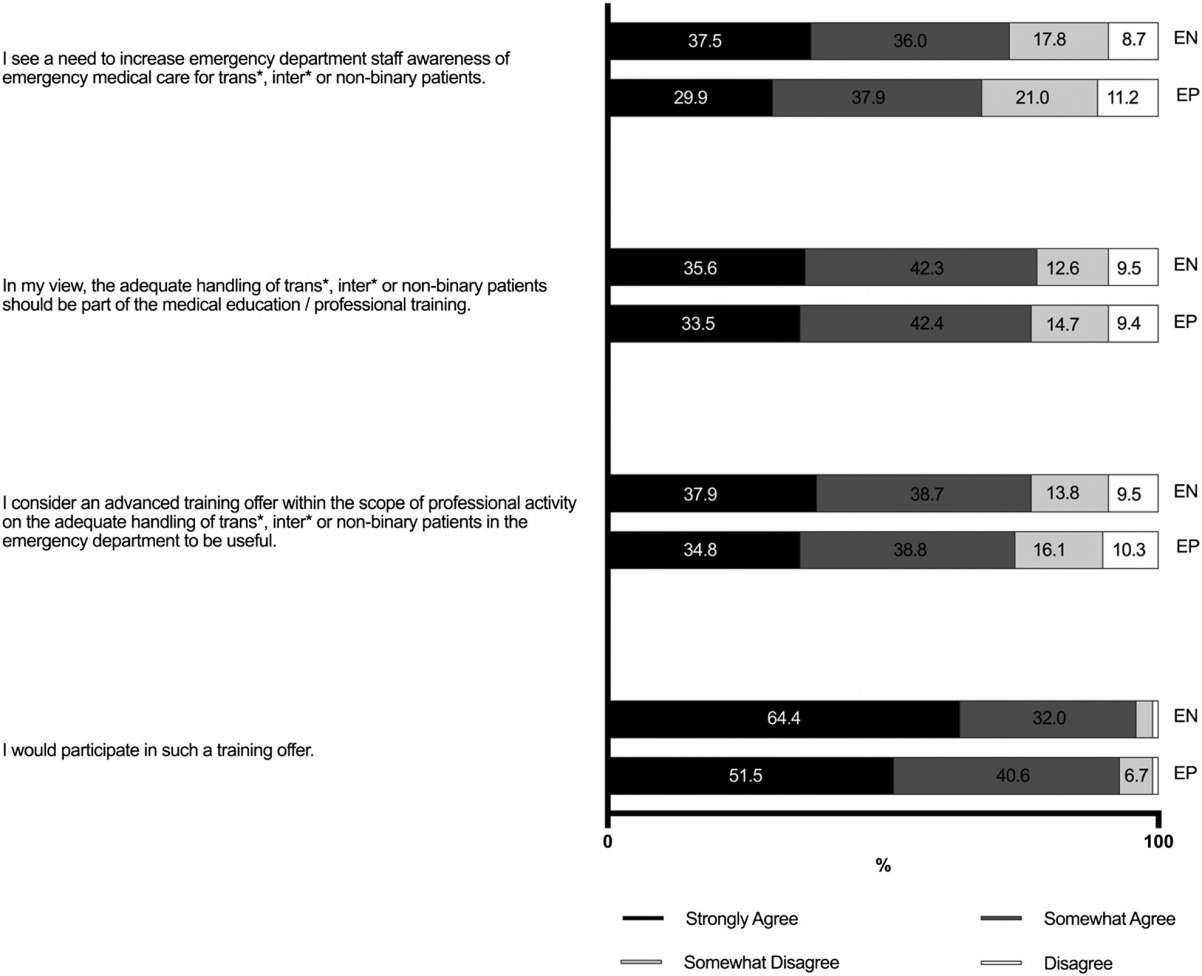
Education and training needs of emergency physicians and nurses in Germany regarding the care of transgender, intersexual, and non-binary patients. *EP*, emergency physician; *EN*, emergency nurse.

Both EPs (68%) and ENs (74%) agreed that there was a need to increase ED staff awareness of emergency medical care for TIN patients, and that this should be part of medical education/professional training (78% of ENs and 76% of EPs). Correspondingly, the majority of both groups believe that continuing medical education (CME) regarding the appropriate management of TIN patients was useful (77% of ENs and 74% of EPs) and that they would participate in such CME when offered (96% of ENs and 92% of EPs) (Figure 4).

## DISCUSSION

In this cross-sectional study, we found that both EPs and ENs in Germany lacked knowledge about important aspects of emergency care of TIN patients and showed differences in attitudes toward communication practices. There was consensus on the need for increased education and training in the management of such patients, with a majority agreeing on the importance of integrating this training into medical education and CME.

Although more than half of the respondents reported encounters with TIN patients in the ED, there was a clear knowledge gap, demonstrated by a lack of awareness of non-medical support services and specific medical regimens related to gender transition. Chisolm-Straker et al reported similar findings in their survey of EPs in the US, showing that the majority of the EPs had seen transgender and gender nonconforming patients, but few had received training in caring for this population and most had inaccurate knowledge of important aspects of transgender and gender nonconforming care.^12^ Steward and O’Reilly pointed out that both nurses and midwives have a wide range of attitudes, knowledge, and beliefs that affect the care they provide to LGBTQ+ patients in general, and many issues of inadequate care appear to be due to a lack of education about LGBTQ health.^16^ In a survey in pediatric EDs in Ireland, Kelleher et al showed that EPs, ENs, and other healthcare workers held positive attitudes towards LGBTQ+ young people; however, they were less confident in their knowledge of specific health issues and self-reported low levels of clinical preparedness.^14^ This lack of knowledge, as demonstrated in studies that included both EPs and ENs, may lead to suboptimal care and increased discomfort for the growing global community of TIN patients as well as healthcare professionals in the ED. Furthermore, the lack of official recommendations for addressing gender-sensitive issues in EDs may contribute to disparities in emergency care experienced by TIN patients.

Several studies have examined the experiences of transgender and gender nonconforming patients in emergency care and demonstrated that more than half of these patients avoid EDs, mostly due to lack of clinician or nurse sensitivity, anticipated discrimination, and fear of mistreatment.^11^ ^,^ ^25^ Focusing specifically on transgender patients, a Swedish study by Carlström et al highlighted the importance of recognizing TIN patients’ vulnerability to violations of dignity, accepting their identity, and focusing on their healthcare needs to restore and maintain their trust in healthcare.^26^ Contrary to these patient reports, and similar to the findings of Chisolm-Straker et al and others, the majority of respondents in this study (both EPs and ENs) expressed comfort in asking TIN patients for their correct form of address and agreed with the importance of documenting both gender identity and biological sex at birth.^11^ ^,^ ^12^ ^,^ ^13^ The reasons for these discrepancies between patients’ experiences and EPs/ENs’ perspectives are not entirely clear and require further investigation.

There may be a discrepancy between the comfort level of asking TIN patients for the correct form of address and the use of the correct pronoun in the actual patient encounter. In addition, most of the participants in this study were younger professionals working and living in large cities in Germany, who may be more open to diverse identities and, therefore, more accustomed to addressing such issues in their professional practice (see Table 1). The art of communication also requires further attention, especially regarding the correct use of gender-sensitive language and respectful communication practices, which should be trained regularly.

The majority of EPs and ENs in this study were aware that TIN individuals are less likely to seek emergency medical care than non-TIN individuals due to concerns about discrimination, indicating an awareness of oppression among EPs and ENs. This empathic awareness of the existence and impact of oppression against TIN people is crucial to removing barriers to healthcare access for these individuals.^27^

Comparing the two groups of EPs and ENs, we found notable differences in existing attitudes toward communication with TIN patients. The ENs were more likely than EPs to limit their communication with TIN patients to what was necessary, out of concern about saying something wrong, and to avoid asking TIN patients questions about genital tract problems. These differences may reflect different roles in patient care but may also reflect different levels of confidence in communicating with TIN patients, highlighting the importance of targeted education and training programs for both EPs and ENs.

In accordance with this finding, most respondents in both groups reported a lack of formal training in the appropriate management of TIN patients, with only a small percentage having received such training during their undergraduate or postgraduate education. These findings are in line with previously published studies from both the US and Europe, highlighting the importance of integrating TIN-specific content into medical and nursing school curricula and providing ongoing CME opportunities for practicing healthcare professionals.^28^ ^,^ ^29^ ^,^ ^30^ In Germany, the InTraHealth self-learning platform for health professionals has been available online since April 2023, offering guidance on addressing and preventing discrimination against TIN individuals in healthcare settings.^31^ However, it is not specifically tailored to EPs and ENs. Similar initiatives and programs exist in the US and Canada but are also often limited to single-session interventions that focus primarily on attitudes and awareness rather than the development of clinical competencies.^32^ Encouragingly, this study reveals a strong desire among EPs and ENs for more education and training to increase ED staff awareness of emergency medical care for TIN patients.

## LIMITATIONS

We used a novel, non-validated survey instrument that relied on self-reported data from EPs and ENs, which may have been subject to response bias, including social desirability bias. In addition, no information was available on non-responders, which introduced the potential for positive selection bias. Emergency department directors, EPs, and ENs with greater interest or comfort with the topic may have been more likely to forward the invitation to their team members or participate in the study, whereas discomfort or lack of familiarity with caring for TIN patients may have acted as potential barriers to participation. In addition, the lack of a reminder for the study invitation may have contributed to a lower response rate, which could affect the generalizability of the findings. Finally, the sample was limited to EPs and ENs in Germany, which could limit the generalizability of the findings to other countries or healthcare systems.

## CONCLUSION

This study adds to the growing body of evidence that emergency physicians have deficits in knowledge and clinical preparedness to care for TIN patients. For emergency nurses, similar deficits seem to exist, exacerbated by greater uncertainty about communicating with patients who identify as transgender, intersexual, and non-binary. This study indicates the need for enhanced education, training, and institutional support to improve the emergency care of TIN patients in Germany. This includes both knowledge of specific treatments and training in sensitive, respectful communication, as well as an understanding of the wider social challenges faced by TIN individuals. By addressing these challenges both in medical/nursing schools and beyond, we can strive to ensure that TIN individuals receive equitable and respectful medical care.
